# Naked-Eye and On-Site Detection of *Staphylococcus aureus* via DNAzyme-Assisted Colorimetric Bioassay for CRISPR/Cas12a

**DOI:** 10.3390/foods15030579

**Published:** 2026-02-05

**Authors:** Xinxin Liu, Ruoxuan Gao, Ruotong Wang, Zhiqiang Xiong, Guangqiang Wang, Lianzhong Ai

**Affiliations:** 1 Shanghai Engineering Research Center of Food Microbiology, School of Health Science and Engineering, University of Shanghai for Science and Technology, Shanghai 200093, China; 2 Department of Food Science & Technology, School of Agriculture and Biology, Shanghai Jiao Tong University, 800 Dongchuan Road, Shanghai 200240, China

**Keywords:** CRISPR/Cas12a, rapid detection, DNAzyme, visualization, *Staphylococcus aureus*

## Abstract

Dairy products have become a key part of the population’s diet due to their nutritional richness, but with increasing demand and market expansion, concerns about the quality and safety of these products have intensified, drawing public attention to the potential risks involved. Their nutritional properties are conducive to the growth of microorganisms, which can lead to contamination with pathogenic bacteria and foodborne diseases. As a common foodborne pathogen, *Staphylococcus aureus* is one of the main causes of foodborne diseases worldwide, and it is also a major source of the contamination of dairy products, posing a serious threat to human health. Although the traditional microbial culture method is accurate, it is cumbersome and time-consuming and requires a sterile environment and large-scale equipment, making it difficult to detect bacteria rapidly. Therefore, the development of convenient, accurate, and sensitive on-site detection methods is essential. In this study, we combined CRISPR/Cas12a technology and DNAzyme colorimetric signal output to design a naked-eye output instant detection platform for *S. aureus*, which can realize the naked-eye reading of the test results. After system optimization, our detection method achieved a detection limit of 10^0^ CFU/mL for pure *Staphylococcus aureus* culture, with a linear range of 10^0^–10^8^ CFU/mL (R^2^ = 0.908). This method exhibits good specificity and can accurately identify *Staphylococcus aureus* and other common foodborne bacteria (*Salmonella*, *Escherichia coli*, *Listeria monocytogenes*, *Lactobacillus plantarum*). It is crucial that when applied to artificially contaminated milk and milk beverages, this method still maintains a detection limit of 10^0^ CFU/mL, demonstrating its strong performance in complex food matrices without the need for complex DNA extraction. This CRISPR/Cas12a DNAzyme colorimetric bioassay can quickly (<2 h) and visually interpret results, providing a powerful, low-cost, and field-deployable tool for enhancing food safety monitoring.

## 1. Introduction

*Staphylococcus aureus* (*S. aureus*) is a typical Gram-positive bacterium [1,2], with physiological and biochemical characteristics of coagulase and catalase positivity [3,4,5] and both salt tolerance and hemolytic activity, and it is easily distinguished from other *staphylococci* by the formation of characteristic golden-yellow colonies on nutrient agar [6,7,8]. The pathogenicity of this bacterium stems from the synergistic action of toxins and invasive enzymes, in which the heat-resistant nuclease encoded by the *nuc* gene serves as a key virulence factor, destroying the immune barrier by degrading the host’s extracellular nucleic acids and facilitating the diffusion of the bacterial invasion, and the high degree of conservatism of the *nuc* gene makes it one of the specific target genes commonly used in detecting and characterizing *S. aureus* [9,10,11]. China’s National Standard for Food Safety (GB29921-2021 [12]) clearly stipulates that the bacteria should not be detected in liquid dairy products. As an important foodborne pathogen, *S. aureus* is prone to contaminating the dairy production chain [13,14,15], and its growth is inhibited at low temperatures, but it can still retain virulence. Under appropriate conditions, strains carrying virulence genes can proliferate rapidly and produce toxins, leading to food poisoning [16,17].

The traditional method of detecting pathogens is the gold standard, but it has limitations such as cumbersome and time-consuming operation [18,19,20]. Emerging molecular biology methods and immunological assays are powerful alternatives to traditional culture methods, with the advantages of accuracy, rapidity, sensitivity, and reproducibility [21,22,23]. However, there are still shortcomings such as the need for specialized technical personnel to operate, complexity of operation, and high cost [24]. Therefore, it is important to develop rapid, low-cost, and field-ready methods for the detection of pathogens.

Currently, clusters of regularly interspaced short palindromic repeat sequence (CRISPR)-associated protein (CRISPR/Cas) systems, such as Cas9, Cas12, and Cas13 [25,26,27], are regarded as next-generation detection technologies [28], and they have been widely designed and applied to detect bacteria [29], viruses [30], metal ions [31], and many other analytes, showing broad prospects in biological detection and analysis.

CRISPR/Cas12 technology accurately recognizes single-base mutations with high sensitivity and specificity [32]. Upon target DNA activation, the technology exhibits non-specific cleavage activity (i.e., trans-cleavage activity) on single-stranded DNA (ssDNA). By co-labeling fluorescein (e.g., FAM) with a fluorescence quencher (e.g., BHQ1) on ssDNA as a reporter molecule, the target can be efficiently detected, and a sensitive fluorescent signal response can be achieved [33]. DNAzymes are a special class of ssDNA sequences that can fold to form a unique three-dimensional structure and exhibit enzyme-like catalytic activity, thus attracting much attention in the field of bioassays. Among them, a DNAzyme based on the G-quadruplex structure, which is a four-stranded structure formed by folding guanine-rich DNA sequences in the presence of metal ions such as K^+^ or Na^+^, is particularly attractive and has high stability [34]. In the presence of metal ions, the G-quadruplex binds to heme, which mimics peroxidase activity [35] and catalyzes the visual signal output of the indicator [36,37], a property that gives it great potential in the field of bioassays.

This study aims to solve the problem of the rapid on-site detection of pathogenic bacteria in complex food matrices. For the first time, a cascade signal amplification colorimetric biosensing platform based on target-responsive DNAzyme was constructed. Unlike traditional methods that rely on antibodies or precision instruments, this platform achieves high-specificity recognition through rationally designed DNAzymes and directly converts their efficient cleavage activity into significant color changes visible to the naked eye, cleverly bypassing the dependence on complex equipment and operations. Of particular importance is that the system effectively suppresses the interference of complex matrices in dairy products in its design, successfully achieving the rapid and highly reliable detection of *Staphylococcus aureus* in actual samples. Therefore, this work not only provides a powerful innovative tool for the on-site monitoring of foodborne pathogens but also offers new design ideas and practical examples for the development of next-generation biosensors for real-time detection.

## 2. Materials and Methods

### 2.1. Strain and Sample Preparation and Genome Extraction

*S. aureus* (CICC 10384), which was kept frozen in a −80 °C refrigerator, was thawed on ice and then inoculated into sterile BHI liquid medium at 1% inoculum and incubated at 37 °C overnight. A total of 10 μL of the bacterial solution was used on the sterile BHI solid medium to mark a line, inverted in an incubator at 37 °C for 24 h, and then placed at 4 °C for short-term storage after the growth of single colonies.

In this study, artificially contaminated dairy samples were prepared using milk and milk beverages purchased from local supermarkets in Shanghai. First, a plate counting method was used to determine the concentration of *S. aureus*, and a bacterial solution of known concentration was prepared. Then, a specific concentration of *S. aureus* bacterial solution was added to the dairy samples so that the samples contained a set amount of *S. aureus*. Under aseptic operating conditions, 1 mL of artificially contaminated dairy samples was placed in a sterilized centrifuge tube and centrifuged at 12,000 rpm for 2 min to remove the supernatant. Next, 1 mL of sterilized deionized water was added, and after resuspension by pipetting and blowing, the supernatant was centrifuged again under the same conditions and discarded. After that, 100 μL of sterilized deionized water was added to resuspend the samples, and the samples were boiled at 100 °C for 10 min to lyse the cells. Finally, the samples were centrifuged again, and the supernatant was collected as a template for subsequent experiments. The supernatant obtained from extraction is the crude genomic DNA template. A microspectrophotometer (NanoDrop One, Thermo Fisher Scientific, Inc., Waltham, MA, USA) was used to determine the concentration and purity of template DNA, and some samples were taken to verify their integrity through 1% agarose gel electrophoresis. DNA templates that met quality requirements were used for subsequent experiments. To control pollution, negative controls and template-free controls were set up in all subsequent CRISPR/Cas12a tests.

### 2.2. Preparation of crRNA

In the CDS sequence of the *S. aureus nuc* gene, 5′-TTTN was selected as the PAM sequence, and the 20 nt DNA sequence after the PAM sequence was used as the spacer sequence of crRNA, which, together with the scaffold sequence, constituted the complete crRNA. The transcription primers and crRNA sequences are shown in Table 1. crRNA was synthesized by the in vitro transcription of T7, followed by purification using the magnetic bead method. The transcription system was prepared according to the instructions of the crRNA Synthesis and Purification Kit (Shanghai, China; Shanghai Tulu Harbor Biotechnology Co., Ltd.), and the transcription template was prepared by reacting at 95 °C for 5 min and then slowly reducing it to room temperature at a rate of 0.2 °C/s. The transcription template was then purified by the magnetic bead method. Then reagents were added according to the order of the instructions, the in vitro transcription reaction system was configured, and the solution was mixed thoroughly and incubated at 37 °C for 4 h. After incubation, the DNA template was removed from the system using DNase I reaction solution. After incubation, the magnetic beads were mixed with crRNA and incubated at room temperature to fully bind to crRNA and rinsed with ethanol to remove impurities, and then the crRNA was eluted with nucleic acid-free purified water. Finally, the purified crRNA was treated at 70 °C for 10 min to inactivate DNase I residues.

### 2.3. Design of PCR, RAA Primers, Fluorescent Probes, and CatG4R Probes

The basic RAA nucleic acid amplification reagent (freeze-dried) was purchased from Shanghai Huicheng Biotechnology Co., Ltd. (Shanghai, China); 2 × Phanta Max Master Mix (Dye Plus) was purchased from Nanjing Novyzan Biotechnology Co., Ltd. (Nanjing, China); and the AxyPrep PCR cleaning kit was purchased from Aisijin Biotechnology (Hangzhou) Co., Ltd. (Hangzhou, China).

The specific procedure for RAA amplification was performed as follows: First, 36 μL of nuclease-free water was added to a lyophilized enzyme pellet. Then, 2 μL each of the upstream and downstream primers (10 μM Nuc-RAA-F and 10 μM Nuc-RAA-R, respectively) were added and mixed thoroughly. Subsequently, 10 μL of the amplification template and a magnesium acetate pellet were added to the mixture, which was inverted several times for complete homogenization. The total reaction volume is 50 μL. Finally, the reaction tube was incubated at 37 °C for 30 min.

### 2.4. DNAzyme Colorimetric Assay

The reaction system of CRISPR/Cas12a (Shanghai, China; Shanghai Tulugang Biotechnology Co., Ltd.) was as follows: 1 × HOLMES Buffer, 250 μM Cas12a, 250 μM crRNA, 250 nM ssDNA reporter, and 10 nM target DNA, for a total volume of 20 μL. The ssDNA reporter in the trans-cut reaction system was replaced with CatG4R. A total of 20 μL of the above Cas12a reaction product was mixed with 10 μM CatG4 solution and annealing buffer (Shanghai, China; Shanghai Yuanye Biotechnology Co., Ltd.), boiled at 100 °C for 5 min, and cooled to room temperature, and then CatG4 and probe CatG4R were annealed. Next, 2 μL of 100 μM hemin (Shanghai, China; Shanghai Titan Technology Co., Ltd.) and 72 μL of MES buffer (0.1 M, pH 4.7, Shanghai Titan Technology Co., Ltd.) were added to the annealing solution and incubated for 30 min in the dark. A total of 2 μL of ABTS (Shanghai Yuanye Biotechnology Co., Ltd.) solution was added to the solution, and finally 2 μL H_2_O_2_ (Shanghai Titan Technology Co., Ltd.) (*v*/*v* = 3%) was added to the solution. A color change was observed, and the absorbance value was measured by a microplate reader.

All reactions were carried out at 37 °C with a total volume of 20 μL for 90 min, and fluorescence was measured by a microplate reader. All key experiments, including bacterial culture, nucleic acid amplification, CRISPR/Cas12a detection, and analysis of spiked samples, were performed with at least three independent biological replicates. Measurements for fluorescence and absorbance readings within each experiment were conducted in triplicate technical replicates. Data are presented as the mean ± standard deviation.

### 2.5. Statistical Analysis

Differences in fluorescence intensity or absorbance values between experimental groups (e.g., target vs. control, different bacterial concentrations) were evaluated for statistical significance using Student’s *t*-test. A *p*-value of less than 0.05 was considered statistically significant. The sensitivity of the assay was assessed by performing linear regression analysis on the measured signal versus the logarithm of bacterial concentration, and the coefficient of determination (R^2^) was calculated. Data are presented as the mean ± standard deviation (SD) from at least three independent experiments.

## 3. Results and Discussion

### 3.1. Principle and Feasibility Validation of DNAzyme-Assisted CRISPR/Cas12a for S. aureus Detection

#### 3.1.1. Experimental Principle

**Figure 1:**
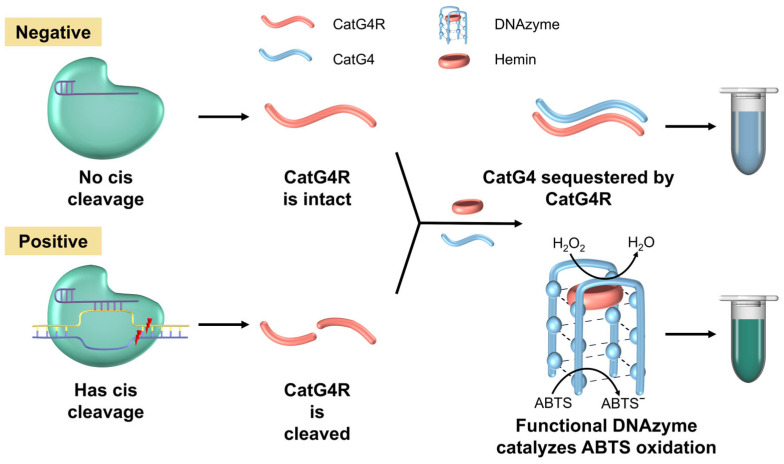
The principle of the DNAzyme-assisted colorimetric bioassay for CRISPR/Cas12a: CatG4R serves as a trans-cleavage substrate in the Cas12a reaction. In negative Cas12a reactions, abundant CatG4R anneals with CatG4, blocking DNAzyme formation and keeping the solution colorless. In positive reactions, Cas12a cleaves CatG4R, freeing CatG4 to combine with hemin and form active DNAzyme, turning the solution blue-green.

In this study, we constructed a colorimetric bioassay (Figure 1) based on CRISPR/Cas12a coupled with DNAzyme for the detection of *S. aureus*, which utilizes the DNAzyme-catalyzed colorimetric reaction to assist the reading of the detection results by the CRISPR/Cas12a system. The core principle is to design guanine (G)-rich ssDNA as a functional probe that self-assembles to form a DNAzyme complex with peroxidase activity upon specific binding to hemin via a G-quadruplex (CatG4) structure. During the detection process, the specific cleavage of the antisense probe CatG4R by the CRISPR/Cas12a system is a key node of system activation: upon the presence of the target, the trans-cutting activity of Cas12a effectively cleaves the CatG4R probe, thereby fully exposing single-strand CatG4 and facilitating its binding to hemin to form an active DNAzyme, which subsequently catalyzes the ABST-H_2_O_2_ reaction. The ABST-H_2_O_2_ chromogenic system generates characteristic green coloration (ABST-); in contrast, the uncut CatG4R probe forms a double-stranded structure with CatG4, resulting in the failure of the G-quadruplex conformation. This leads to the closure of the hemin-binding site, resulting in the loss of catalytic activity and the retention of a colorless background in the solution.

#### 3.1.2. Feasibility Study

To validate the feasibility of DNAzyme-assisted colorimetric bioassay for CRISPR/Cas12a for nucleic acid detection, a series of experiments was carried out. Firstly, *S. aureus* genomic DNA was extracted and subsequently detected using the CRISPR/Cas12a system, with the probe replaced with CatG4.

**Figure 2:**
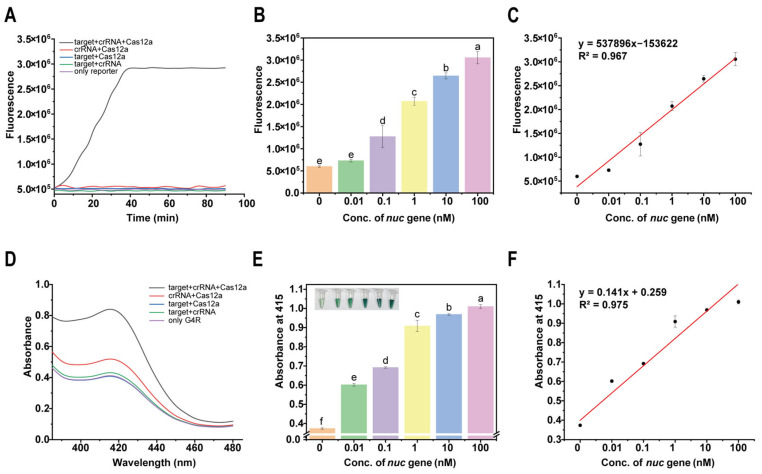
Feasibility validation of DNAzyme-assisted colorimetric bioassay for CRISPR/Cas12a: (**A**) validation of trans-cleavage activity of fluorescent signal; (**B**) fluorescence intensity at reaction time of 90 min; (**C**) linear relationship between fluorescence intensity and concentration of *nuc* gene; (**D**) validation of inverse cutting activity of colorimetric signal; (**E**) absorbance value at reaction time of 90 min; (**F**) linear relationship between absorbance value and *nuc* gene concentration. From a to f, the significance gradually decreases, and there is no significant difference between the same letters.

When the amplified nucleic acid sequence was successfully recognized by the specific crRNA, the trans-cleavage activity of Cas12a was activated, leading to the non-specific degradation of the fluorescent reporter probe. To verify the trans-cleavage activity of CRISPR/Cas12a, four sets of variable experiments were designed. The experiments were performed by the fluorescence method for signal output, and the fluorescence intensity of each group was detected within 90 min using an enzyme marker. The results were plotted based on the fluorescence signal kinetics. The experimental group contained all necessary components for fluorescence cleavage, while the four negative control groups included the Cas12a-free group, the crRNA-free group, the target DNA-free group, and the fluorescent reporter probe-only group. As shown in Figure 2A, only the experimental group exhibited an increase in fluorescence intensity over time, while the fluorescence values of the four negative control groups remained relatively constant. At 40 min, the reaction has basically reached the endpoint (Figure 2A). If the reaction time is continually increased, the fluorescence value will not change. At this point, since the reaction has reached the endpoint, we will use 40 min as the reaction time in the future. This result fully indicates that Cas12a exhibits the trans-cleavage activity of the non-specific cleavage of the ssDNA reporter inspired by the specific recognition of target DNA. In the absence of specific crRNA or target DNA, the trans-cleavage activity of Cas12a is dormant, preventing the degradation of the fluorescent reporter probe and quenching the fluorescence signal. However, when target DNA, crRNA, and the Cas12a protein are all present, the fluorescent reporter probe is degraded, resulting in a fluorescent signal. Further analysis revealed that fluorescence intensity increased in a concentration-dependent manner (Figure 2B) and demonstrated a strong linear correlation with target DNA concentration (R^2^ = 0.967, Figure 2C).

Compared to fluorescence signal detection, colorimetric signal output is simpler and does not require complex instrumentation. In this study, a colorimetric bioassay technique based on DNAzyme-assisted CRISPR/Cas12a was successfully developed and tested. When target DNA is present, CatG4R is cleaved by the activated Cas12a, which leads to the formation of DNAzyme. The DNAzyme catalyzes the conversion of ABTS to ABST-, resulting in a distinct color change and specific absorption at 415 nm. In contrast, in the absence of target DNA, CatG4R remains intact and is unable to catalyze a reaction with ABTS. In the presence of target DNA, crRNA, and Cas12a, a significant color change was observed, and the absorbance at 414 nm was significantly higher (Figure 2D). Furthermore, absorbance increased with an increase in target DNA concentration, showing a strong linear correlation (R^2^ = 0.975, Figure 2E,F).

Experimental group: 1 × HOLMES buffer, 250 nM Cas12a, 250 nM crRNA, 250 nM ssDNA fluorescent reporter, 10 nM target DNA.

Control group 1 (excluding Cas12a): 1 × HOLMES buffer, 250 nM crRNA, 250 nM ssDNA fluorescent reporter, 10 nM target DNA.

Control group 2 (without crRNA): 1 × HOLMES buffer, 250 nM Cas12a, 250 nM ssDNA fluorescent reporter, 10 nM target DNA.

Control group 3 (without target DNA): 1 × HOLMES buffer, 250 nM Cas12a, 250 nM crRNA, 250 nM ssDNA fluorescent reporter.

Control group 4 (reporter only): 1 × HOLMES buffer, 250 nM ssDNA fluorescence reporter.

### 3.2. Optimization of Assay Conditions for DNAzyme-Assisted Colorimetric Bioassay for CRISPR/Cas12a

To achieve optimal detection results, this study meticulously optimized the reaction conditions for the DNAzyme-assisted colorimetric bioassay for CRISPR/Cas12a. To minimize false positives and improve accuracy and reliability, optimization experiments were conducted on the concentrations of the antisense probe CatG4R Cas12a protein.

**Figure 3:**
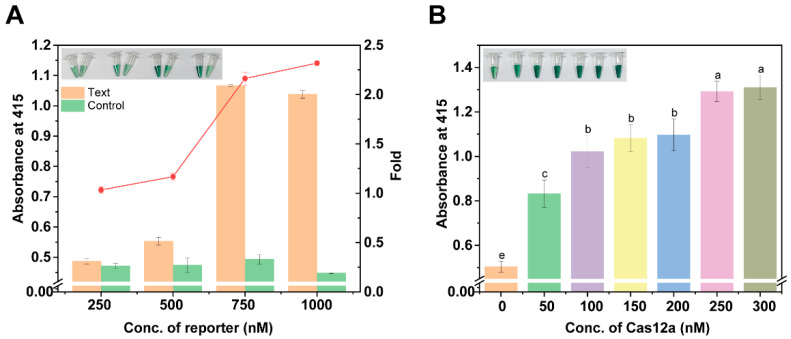
Optimization of assay conditions for DNAzyme-assisted colorimetric bioassay for CRISPR/Cas12a: (**A**) optimization of CatG4R reporter concentration; (**B**) optimization of Cas12a protein concentration. For different letters, the significance gradually decreases, and there is no significant difference between the same letters.

The optimization of antisense probe CatG4R concentration is shown in Figure 3A. By visually assessing the color depth difference between the negative and positive groups, the differentiation between the two groups at varying concentrations can be initially determined. Within the tested concentration range of 250–1000 nM, the most pronounced color difference between the negative and positive groups was observed at a CatG4R concentration of approximately 1000 nM, suggesting that this concentration may provide an optimal balance between assay sensitivity and specificity. Absorbance was further quantified using an enzyme marker, and the results confirmed that, at a CatG4R concentration of 1000 nM, the false positive signal was the weakest in the negative group and stronger in the experimental group. The signal-to-noise ratio was maximized, with the absorbance in the experimental group being 2.32 times higher than that in the negative group. This finding indicates that a CatG4R concentration of 1000 nM effectively differentiates between negative and positive samples, minimizes the false positive rate, and thus identifies a CatG4R concentration of 1000 nM as the optimal condition for subsequent studies.

The optimization of Cas12a protein dosage is shown in Figure 3B. Within the tested concentration range of 0–300 nM, the color intensity of the solution gradually increased as the Cas12a protein dosage was elevated. This could be attributed to the fact that the increase in Cas12a protein concentration enhances the reaction, leading to more degradation of the substrate and, consequently, more pronounced color changes. When the Cas12a protein concentration reached 250 nM, the reaction was relatively more adequate, and the experimental phenomenon was more obvious, which indicated that at this concentration, the Cas12a protein could effectively perform its function and promote the detection reaction. Therefore, a Cas12a protein concentration of 250 nM was selected as the optimal condition for subsequent experiments to ensure the adequacy and accuracy of the detection reaction. The optimization of the reaction conditions outlined above provides a solid foundation for the further research and application of the DNAzyme-assisted colorimetric bioassay for CRISPR/Cas12a.

### 3.3. Sensitivity and Specificity Characterization of DNAzyme-Assisted Colorimetric Bioassay for CRISPR/Cas12a

#### 3.3.1. Sensitivity Characterization

**Figure 4:**
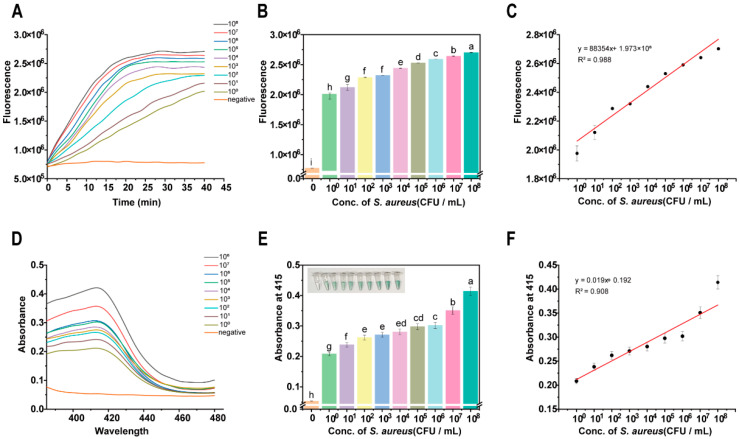
Sensitivity of DNAzyme-assisted colorimetric bioassay for CRISPR/Cas12a for detecting *S. aureus*: (**A**) kinetic characterization of fluorescence signal; (**B**) fluorescence intensity at reaction time of 40 min; From a to i, the significance gradually decreases, and there is no significant difference between the same letters. (**C**) linear relationship between fluorescence intensity and *S. aureus* concentration; (**D**) full-spectrum scanning of colorimetric signal; (**E**) absorbance value at 415 nm; From a to h, the significance gradually decreases, and there is no significant difference between the same letters. (**F**) linear relationship between absorbance value and *S. aureus* concentration.

After optimizing the reaction conditions, this study investigated the performance of the DNAzyme-assisted colorimetric bioassay for CRISPR/Cas12a for the detection of *S. aureus*. Since the fluorescence assay is most widely used in CRISPR/Cas12a detection, this study first used the colorimetric assay to detect *S. aureus*. Based on the screening of crRNA in Table 1, Cas12a can be guided to complete trans-cleavage within 40 min. So the fluorescence intensity of each group within 40 min was detected using an enzyme-labeled method, and the results are shown in Figure 4A–C. Figure 4A displays the real-time fluorescence detection of *S. aureus* based on CRISPR/Cas12a. As the concentration of *S. aureus* colonies increased, the fluorescence signal was strengthened correspondingly, which suggests that the CRISPR/Cas12a system is capable of effectively recognizing and responding to different concentrations of *S. aureus*. Further analysis demonstrated that a strong linear relationship was obtained between the fluorescence signal and the concentration of *S. aureus* colonies in the range of 10^0^–10^8^ CFU/mL, with a high linear correlation coefficient of R^2^ = 0.988, as shown in Figure 4B,C. The corresponding fluorescence intensity increased from 2.0 × 10^6^ RFU at 10^0^ CFU/mL to 2.75 × 10^6^ RFU at 10^8^ CFU/mL. In addition, the detection limit of the fluorescence readout method for *S. aureus* was 10^0^ CFU/mL, which provided an important reference for the subsequent optimization of the detection method.

To meet the need for visualization and simplicity of the assay, a DNAzyme-assisted colorimetric bioassay for CRISPR/Cas12a was used for the detection of *S. aureus*. After the amplification and detection of different concentrations of *S. aureus*, distinct color changes were observed, as shown in Figure 4D. As the concentration of *S. aureus* increased, more CatG4R was degraded to generate DNAzyme. This DNAzyme subsequently catalyzed an enhanced ABTS-H_2_O_2_ reaction, resulting in a more pronounced color change. The corresponding absorbance spectra and absorbance at 415 nm are shown in Figure 4E. The absorbance at 415 nm showed a concentration-dependent increase with the concentrations of *S. aureus*, which indicates that the colorimetric bioassay has a good quantitative capacity for the detection of *S. aureus*. Further analysis confirmed that within the 10^0^–10^8^ CFU/mL range, absorbance and *S. aureus* colony concentration exhibited a strong linear relationship (R^2^ = 0.908) (Figure 4F).

In order to further evaluate the analytical sensitivity of the assay, the detection limit (LOD) and quantification limit (LOQ) were determined according to the IUPAC guidelines. The LOD is defined as the concentration corresponding to the average signal of the blank sample plus three times its standard deviation, calculated as 5.6 CFU/mL. The LOQ is defined as the concentration corresponding to the average value of the blank signal plus ten times its standard deviation, resulting in 18.7 CFU/mL. These values confirm the reliable detection ability of the assay, which is far below the lower limit of the linear range.

We studied the potential bias in extreme situations. At concentrations close to or below the quantification limit (<20 CFU/mL), although detectable signals above the background can be observed, the signal-to-noise ratio decreases, and the coefficient of variation between replicates increases. Therefore, 10^2^ CFU/mL was established as the actual lower limit for reliable quantification to ensure accuracy and precision. At a high concentration of 10^8^ CFU/mL, no signal saturation or linear deviation was observed. The absorbance value remains within the reliable detection range of the microplate reader, and this data point is in good agreement with the calibration curve (Figure 4F), indicating that its dynamic range is wide and suitable for detecting high-level pollution.

#### 3.3.2. Specific Characterization

**Figure 5:**
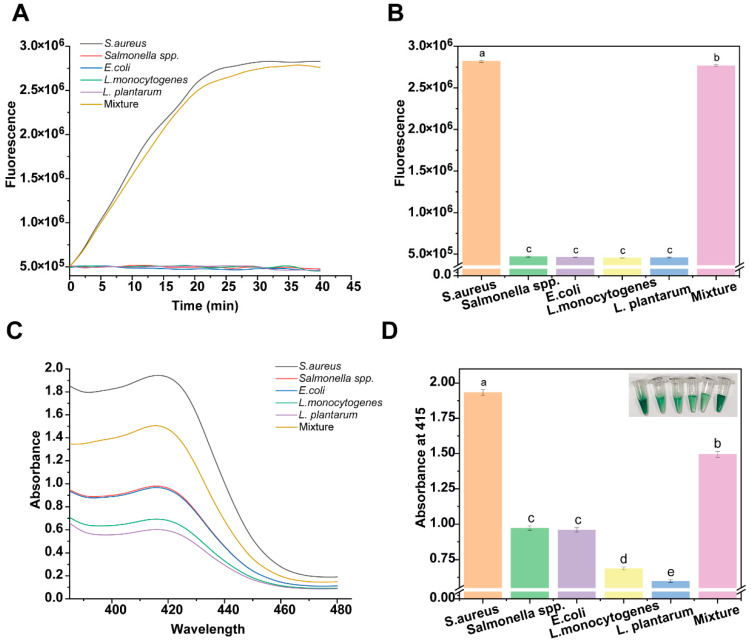
DNAzyme-assisted colorimetric bioassay for CRISPR/Cas12a for detecting specificity of *S. aureus* against other common bacteria: (**A**) kinetic characterization of fluorescence signal; (**B**) fluorescence intensity at reaction time of 40 min; From a to c, the significance gradually decreases, and there is no significant difference between the same letters. (**C**) full-spectrum scanning of colorimetric signal; and (**D**) absorbance value at 415 nm; From a to e, the significance gradually decreases, and there is no significant difference between the same letters.

As shown in Figure 5A,B, the fluorescence signals of four interfering bacteria, *Salmonella*, *Escherichia coli* (*E. coli*), *Listeria monocytogenes*, *Lactobacillus plantarum* (*L. plantarum*), and *Lactobacillus plantarum* (*L. plantarum*), were significantly lower than those of *S. aureus* samples. Notably, in both pure *S. aureus* samples and *S. aureus* mixed with these interfering bacteria (labeled “Mixture”), a significant increase in fluorescence signals was seen. This demonstrates that the method has good specificity and that *S. aureus* can be effectively detected even in the presence of interfering bacteria.

When the DNAzyme-assisted colorimetric bioassay for CRISPR/Cas12a was further used for detection, the relative absorbance at 415 nm was significantly increased compared to other interfering bacteria as long as *S. aureus* was present, and this result was consistent with the fluorescence assay, as shown in Figure 5C,D. This shows that interfering bacteria have a negligible effect on the DNAzyme-assisted colorimetric bioassay for the CRISPR/Cas12a detection of *S. aureus*. Therefore, the colorimetric bioassay proposed in this study has good selectivity for the detection of *S. aureus* and can effectively distinguish *S. aureus* from other interfering organisms, which provides a reliable method for the accurate detection of *S. aureus*.

### 3.4. Detection of Artificially Contaminated Dairy Products

After successfully applying CRISPR/Cas12a technology to detect *S. aureus* in BHI broth, this study further explored its detection capability in real samples. For dairy products (including milk and milk beverages), the baseline colony concentration was first determined via plate counting. Subsequently, different levels of *S. aureus* were spiked into the samples, which were then detected by conventional fluorescence and DNAzyme-assisted biocolorimetric assays.

#### 3.4.1. Milk Sample Testing

**Figure 6:**
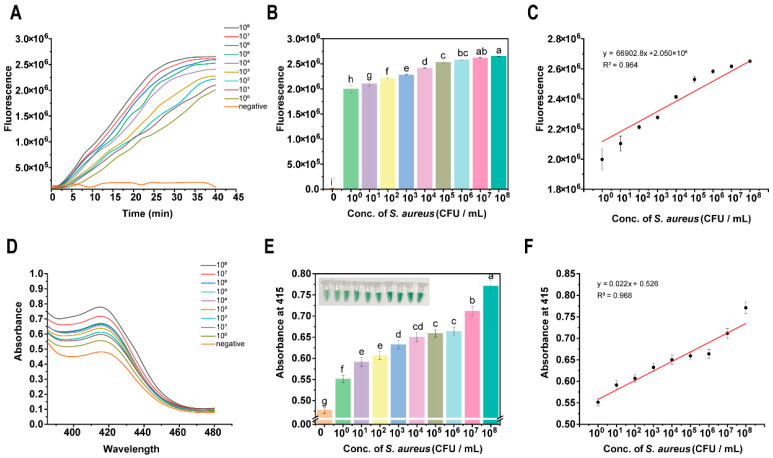
DNAzyme-assisted colorimetric bioassay for CRISPR/Cas12a for detecting *S. aureus* in milk: (**A**) kinetic characterization of fluorescence signal; (**B**) fluorescence intensity at reaction time of 40 min; From a to i, the significance gradually decreases, ab represents significance between a and b, and remains the same thereafter, and there is no significant difference between the same letters. (**C**) linear relationship between fluorescence intensity and concentration of *Staphylococcus aureus*; (**D**) full-spectrum scanning of colorimetric signal; (**E**) absorbance value at 415 nm; From a to g, the significance gradually decreases, cd represents significance between c and d, and remains the same thereafter, and there is no significant difference between the same letters. (**F**) linear relationship between absorbance value and *S. aureus* concentration.

For milk samples, the results of the conventional fluorescence method showed (see Figure 6A–C) that the fluorescence signal was enhanced with increasing *S. aureus* concentration, with a strong linear relationship (R^2^ = 0.939), and the detection limit could reach 10^0^ CFU/mL. This suggests that it has high sensitivity and accuracy for the detection of *S. aureus* in milk. In contrast, the DNAzyme-assisted colorimetric bioassay of CRISPR/Cas12a (Figure 6D,E) revealed that 415 nm absorbance increased with the concentration of *S. aureus*, showing good linearity (R^2^ = 0.921), and the limit of detection was 10^0^ CFU/mL. Thus, this colorimetric bioassay also demonstrates high sensitivity and accuracy for *S. aureus* detection in milk. Both methods showed high sensitivity and accuracy in detecting *S. aureus* in milk.

#### 3.4.2. Milk Beverage Sample Testing

**Figure 7:**
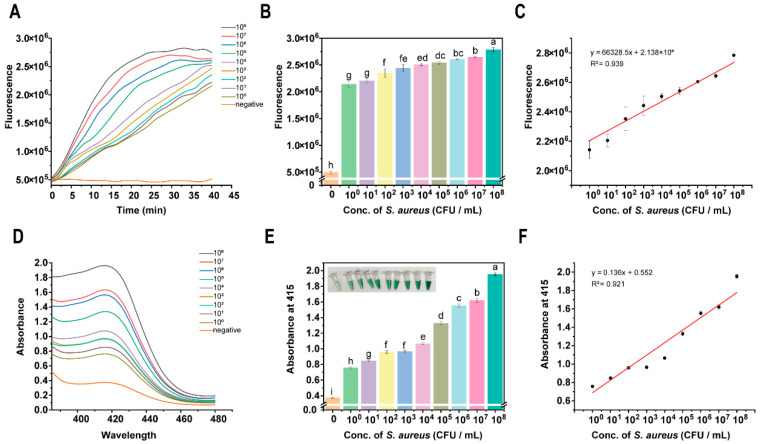
DNAzyme-assisted colorimetric bioassay for CRISPR/Cas12a for detecting *S. aureus* in milk beverages: (**A**) kinetic characterization of fluorescence signal; (**B**) fluorescence intensity at reaction time of 40 min; From a to h, the significance gradually decreases, bc represents significance between b and c, and remains the same thereafter, and there is no significant difference between the same letters. (**C**) linear relationship between fluorescence intensity and concentration of *Staphylococcus aureus*; (**D**) full-spectrum scanning of colorimetric signal; (**E**) absorbance value at 415 nm; From a to i, the significance gradually decreases, and remains the same thereafter, and there is no significant difference between the same letters. (**F**) linear relationship between absorbance value and *S. aureus* concentration.

The conventional fluorescence method also performed well in detecting in milk beverages. The fluorescence signal was gradually enhanced with increasing *S. aureus* concentration, showing significant linearity (R^2^ = 0.964) and a detection limit of 10^0^ CFU/mL (Figure 7A–C). This result further confirms the ability of the conventional fluorescence assay for detecting *S. aureus* in milk beverages. Similarly, the DNAzyme-assisted colorimetric bioassay for CRISPR/Cas12a demonstrated a significant linear relationship between the absorbance at 415 nm and the concentration of *S. aureus* (R^2^ = 0.968), with a detection limit of 10^0^ CFU/mL (Figure 7D,E). This shows that the method is equally sensitive and accurate for the detection of *S. aureus* in milk beverages.

In summary, the method used in this study can rapidly and efficiently detect *S. aureus* in milk and milk beverages at a concentration of 10^0^ CFU/mL and allows for the naked-eye reading of the results. This aligns with the current National Standard for Food Safety (GB 29921-2021), demonstrating the practical application of DNAzyme-assisted colorimetric bioassay for CRISPR/Cas12a for the detection of *S. aureus* in milk and milk beverages and providing a new technological tool for food safety monitoring.

## 4. Conclusions

Although the nutritional cells of *Staphylococcus aureus* may have difficulty proliferating in the acidic or preservative-containing environment of certain beverages, the greatest risk lies in their ability to produce heat-stable enterotoxins. If conditions allow for bacterial growth, these toxins can be pre-formed in the product and remain active even after processes such as pasteurization that kill bacterial cells. Therefore, the ability to sensitively detect even low levels of live *Staphylococcus aureus* before producing large amounts of toxins is crucial for active food safety interventions. This study developed a specific biological colorimetric detection method for *Staphylococcus aureus* by integrating CRISPR/Cas12a technology with a DNA enzyme-catalyzed colorimetric system. The process of this method includes thermal cracking to extract genomic DNA, RAA, CRISPR/Cas12a trans-cleavage activation, and the subsequent DNA enzyme-catalyzed color reaction. This system performs RAA amplification on specific nucleic acid sequences of *Staphylococcus aureus* and utilizes CRISPR/Cas12a for high-precision recognition. The experimental results show that the detection accuracy of this method for target nucleic acids can reach 0.01 nM, which is superior to the traditional fluorescence method (0.1 nM). The detection sensitivity of *Staphylococcus aureus* and its contaminated milk or dairy beverage samples reaches 10^0^ CFU/mL. Even in the presence of multiple interfering bacteria, the system can still accurately identify the target bacteria, demonstrating excellent specificity and anti-interference ability.

While this study demonstrates the high sensitivity and specificity of the CRISPR/Cas12a-DNAzyme assay for detecting *S. aureus* in artificially contaminated dairy products, it is important to acknowledge a key methodological consideration. The use of spiked samples, wherein actively cultured bacteria are introduced into a sterile matrix, serves as a vital and controlled first step for validating the fundamental analytical performance of a new detection platform. However, it may not fully replicate the state and behavior of pathogens in naturally contaminated food. In real-world scenarios, microorganisms can be subjected to various environmental stresses during processing and storage, potentially leading to sublethal injury, entry into a viable but non-culturable state, or integration within biofilms. These physiological changes could affect cell lysis efficiency and the availability of target nucleic acids, thereby potentially influencing detection outcomes. Therefore, the excellent performance reported herein should be interpreted as establishing the robust inherent capability of the assay.

In order to further improve the convenience and integration of its on-site applications, this study can also be combined with emerging functional materials. For example, inspired by intelligent hydrogel research [38], key biochemical reagents (such as crRNA, Cas12a, DNAzyme substrate, etc.) in the detection system can be pre-fixed in the hydrogel or porous membrane matrix and developed into direct dropping solid test strips or test cores. This modular approach is expected to optimize naked-eye reading ability and truly achieve the portable detection of sample input and output results. In addition, the advantage of the CRISPR/Cas12a DNA enzyme cascade detection strategy proposed in this study lies not only in the performance of each component itself but also in the integrated collaborative design of the two core functions of “specific recognition” and “signal transduction output”. The idea of systematically improving overall task performance by integrating different functional units has also been studied in other cutting-edge technological fields. For example, in the research of 6G mobile networks, scholars have proposed a new paradigm called “Integrated Perception and Edge Intelligence (ISEA)”, which focuses on optimizing perception, information processing, and communication functions through collaborative design to achieve the efficient execution of complex tasks [39].

This collectively reveals an important trend in the development of interdisciplinary technology: shifting from discrete functional module stacking to integrated system design and functional collaboration to achieve better overall performance, higher operational efficiency, and better user experience. This study can be seen as a successful practice of this design concept in the field of biomolecular diagnostics, supports naked-eye interpretation, and has significant potential for on-site application.

## Figures and Tables

**Table 1:** Sequences of transcription primers and crRNA for CRISPR/Cas12a-assisted colorimetric detection of *Staphylococcus aureus*.

Sequence Name	Sequence (5′-3′)
Nuc-RAA-F	CTTATAGGGATGGCTATCAGTAATGTTTCG
Nuc-RAA-R	TCTATTTACGCCATTATCTGTTTGTGATGC
CatG4	TGGGTAGGGCGGGTTGGGAAA
CatG4R	TTTCCCAACCCGCCCTACCCA
crRNA-M1	GCAACTAGTAGCGAAAAAGAATCTACACTTAGTAGAAATTACTATAGTGAGTCGTATTA
crRNA-M2	CTTAGACTTGAAACTACAACATCTACACTTAGTAGAAATTACTATAGTGAGTCGTATTA
crRNA-M3	TTCCTGAGCTACTTAGACTTATCTACACTTAGTAGAAATTACTATAGTGAGTCGTATTA
crRNA-M4	TTTTCTACCATTTTTTTCGTATCTACACTTAGTAGAAATTACTATAGTGAGTCGTATTA
crRNA-M5	TCAGTTCTTTGACCTTTCTCATCTACACTTAGTAGAAATTACTATAGTGAGTCGTATTA
crRNA-M6	TACCATTTTTCCATCAGCATATCTACACTTAGTAGAAATTACTATAGTGAGTCGTATTA
crRNA	UAAUUUCUACUAAGUGUAGAUGUUGUAGUUUCAAGUCUAAG

## Data Availability

The original contributions presented in this study are included in the article. Further inquiries can be directed to the corresponding author.
